# mGluR5 protect astrocytes from ischemic damage in postnatal CNS white matter

**DOI:** 10.1016/j.ceca.2015.06.010

**Published:** 2015-11

**Authors:** Ilaria Vanzulli, Arthur M. Butt

**Affiliations:** Institute of Biomedical and Biomolecular Sciences, School of Pharmacy and Biomedical Sciences, University of Portsmouth, U.K.

**Keywords:** Astrocyte, mGluR, Glutamate, Ischemia, Calcium, White matter

## Abstract

Astrocytes perform essential neuron-supporting functions in the central nervous system (CNS) and their disruption has devastating effects on neuronal integrity in multiple neuropathologies. Although astrocytes are considered resistant to most pathological insults, ischemia can result in astrocyte injury and astrocytes in postnatal white matter are particularly vulnerable. Metabotropic glutamate receptors (mGluR) are neuroprotective in ischemia and are widely expressed by astrocytes throughout CNS grey matter, but their potential cytoprotective role in astrocytes had not been determined. Here, we identify functional expression of group I mGluR in white matter astrocytes and demonstrate their activation protects astrocytes from ischemic damage in the postnatal mouse optic nerve. Optic nerve astrocytes are shown to express mGluR5 using immunolabelling of sections and explant cultures from transgenic reporter mice in which GFAP drives expression of EGFP. In addition, using Fluo-4 calcium imaging in isolated intact optic nerves, we show that the group I/II mGluR agonist ACPD and the specific group I mGluR agonist DHPG evoke glial Ca^2+^ signals that were significantly inhibited by the group I mGluR antagonist AIDA. A key finding is that activation of group I mGluR protects astrocytes against oxygen-glucose deprivation (OGD) *in situ*, in isolated intact optic nerves from GFAP-EGFP mice. This study identifies a role for group I mGluR in protecting astrocytes against ischemia in postnatal white matter and suggests this may be a strategy for limiting damage in neuropathologies involving excitotoxity.

## Highlights

• White matter astrocytes are vulnerable to ischemia.
• Immunolabelling and calcium imaging demonstrate mGluR5 in white matter astrocytes.
• Activation of mGluR protects astrocytes against ischemic damage.

## Graphical abstract

**Figure:**
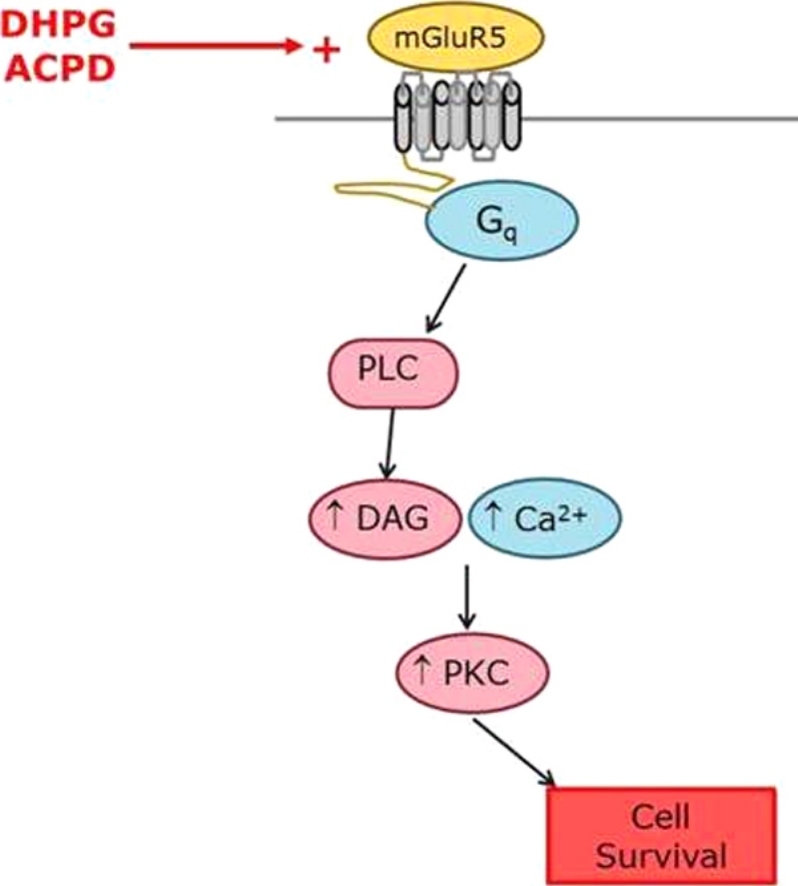

## 1 Introduction

Astrocytes have diverse and important functions in the pathology of cerebral ischemia [1]. Although astrocytes may be less susceptible than neurons to ischemic damage, astrocyte death is an important component of ischemia and has detrimental effects on neuronal survival and integrity [2], [3], [4], [5]. The susceptibility of astrocytes to ischemia depends on the brain region [6], and astrocytes in developing white matter may be particularly vulnerable [5], [7], [8]. Ischemia-mediated cytotoxicty involves multiple events, but there is a key role for raised extracellular glutamate. Glutamate is central to white matter pathology [9], [10], and is excitoxic for astrocytes in vitro [11] and in situ in the spinal cord [12]. The cytotoxic effects of glutamate are mediated largely through AMPA- and NMDA-type ionotropic glutamate receptors (iGluR). In contrast, group I metabotropic GluR (mGluR) have a protective role in neurons and oligodendrocytes exposed to ischaemic injury [13], [14], [15], but astrocytes have been neglected in this context. Group I mGluRs (mGluR1 and mGluR5) are positively coupled to phospholipase C (PLC) and inositol triphosphate (InsP_3_) formation, resulting in release of Ca^2+^ from endoplasmic reticulum (ER) intracellular stores. Group I mGluR have been shown to mediate Ca^2+^ signals in grey matter astrocytes [16], [17] and regulate synaptic activity at the tripartite synapse [18]. White matter astrocytes also display glutamate-mediated Ca^2+^ signaling [19], although the significance of mGluR and their possible role in the astrocyte response to ischemia was unknown. In this study, we demonstrate functional expression of group I mGluR in postnatal white matter astrocytes and show for the first time that they protect astrocytes from ischemia.

## 2 Materials and methods

### 2.1 Animals

Mice aged postnatal day (P)8–15 were used throughout and killed humanely to obtain tissue, in accordance with the UK Animals (Scientific Procedures) Act, 1986. Wild type mice of the C57BL/6 strain were used, or transgenic mice in which the fluorescent reporter enhanced green fluorescence protein (EGFP) is driven by the astroglial gene glial fibrillary acidic protein (GFAP) (gift from Frank Kirchhoff, University of Saarland, Germany). Brain tissue and optic nerves were removed for immunostaining, tissue culture, calcium imaging or ischemia experiments.

### 2.2 Optic nerve explant cultures

Optic nerve explant cultures were prepared as previously described [20]. Briefly, optic nerves from P8 GFAP-EGFP mice were placed directly into 50 μl dissecting medium consisting of high glucose Dulbecco's Modified Eagle Medium (DMEM) (Sigma-D5671) containing 10% Fetal Calf Serum (FCS; Life Technologies), l-Glutamine (Sigma) and 0.1% Gentamycin (Life Technologies). Nerves were finely chopped with a scalpel blade and triturated with pipettes of decreasing diameter. After adding 50 μl dissecting medium, the solution was pipetted onto poly-l-lysine/laminin coated coverslips (1 nerve per coverslip). After 24 h, the dissecting medium was replaced with a low serum (0.5%) modified Bottenstein and Sato (B&S) medium [21]. Explant cultures were treated for immunolabelling after 7–10 days in vitro (DIV).

### 2.3 Immunolabelling

Brain tissue and optic nerves from P8-9 GFAP-EGFP mice were fixed with 4% paraformaldehyde (PFA), and explant cultures with 1% PFA, all containing 15% picric acid; fixation was for 24 h for brain tissue, 1 h for optic nerves and 10 min for explant cultures. Fixed brain and optic nerves were cryoprotected in 30% sucrose at 4 °C for 24–48 h and embedded in Cryo-M-Bed (Bright Instruments Company Ltd), before rapidly freezing and storage at −80 °C until use. Coronal brain sections and longitudinal optic nerve sections (14 μm) were collected onto Polysine^®^ coated slides (Thermo-Scientific). Sections and explant cultures were treated the same subsequently. A blocking stage was performed by incubation in 10% normal goat (NGS) in phosphate buffered saline (PBS) for 1-2 h, then washed 3 times in PBS and incubated overnight with primary antibodies in blocking solution containing 0.01% Triton-X-100: rabbit anti-mGluR5, at 1:1000 (Neuromics); chicken anti-GFAP, at 1:500 (Chemicon). Samples were then washed 3 times in PBS and incubated with the appropriate secondary antibodies conjugated with ^568^Alexafluor (1:400, Life Technologies). Following immunolabelling, coverslips/sections were mounted with Vectasheild^®^ (VectorLabs). Controls were performed in which sections were preabsorbed with antigen peptide overnight prior to incubation in the primary antibody for mGluR5 and no immunolabelling was detected in these sections. Images were acquired using a Zeiss Axiovert LSM710 VIS405 confocal microscope, using multichannel sequential scanning, narrow bandwidths, and minimal laser power and gain to prevent cross-talk between the channels. Immunolabelling results are presented as two-dimensional flattened images of the *z*-stacks, approximately 10–20 *z*-sections of <0.75 μm thickness, and a colocalization channel was generated to illustrate in the voxels in which the two channels overlap with the same intensity (Volocity 6.1, Perkin Elmer).

### 2.4 Calcium imaging

Optic nerves from P8-P13 wild type C57BL/6 mice were isolated intact for Fluo-4 calcium imaging, as described previously [19]. In brief, nerves were incubated for 1 h in *a*CSF containing 4 μM Fluo-4 AM (Molecular Probes); *a*CSF comprised (in mM) 133 NaCl, 3 KCl, 1.5 CaCl_2_, 1.2 NaH_2_PO_4_, 1.0 MgCl_2_, 10 d-glucose, 10 HEPES, pH 7.3. Loaded nerves were then placed in a perfusion chamber under a Zeiss LSM 5 Pascal Axioskop 2 confocal microscope and continuously perfused with *a*CSF via a multitap system. Nerves were imaged using a 20x/0.50 WPh2 Achroplan water immersion lens objective using excitation at 488 nm and optical *z*-sections (7–8 sections at 2–3 μm intervals) were obtained using the Zeiss LSM Image Examiner software (Zeiss, Germany). Regions of interest (ROI) comprising individual glial cell bodies were selected and changes in fluorescence intensity above baseline (Δ*F*/*F*) were measured in arbitrary units (AU). The multitap system allowed rapid turnover of solution bathing the nerve to one containing pharmacological agents, made up in *a*CSF to a final concentration of 100 μM (all agents purchased from Tocris): adenosine triphosphate (ATP); group I/II mGluR agonist ACPD ((±)-1-Aminocyclopentane-*trans*-1,3-dicarboxylic acid); selective group I mGluR agonist DHPG ((*RS*)-3,5-Dihydroxyphenylglycine); group I antagonist AIDA ((*RS*)-1-Aminoindan-1,5-dicarboxylic acid). In each nerve, a brief pulse (30 s) of ATP (100 μM) was used at the beginning and end of the experiment to confirm the viability of the nerve, and in this way the responses to mGluR agonists could be compared to the maximal response observed in ATP, in paired recordings from individual cells [19]. Data were expressed as mean ± SEM, where ‘*n*’ represents the number of cells, and significance was determined by paired t-tests, using Prism 5.0 (Graphpad).

### 2.5 Oxygen-glucose deprivation

Optic nerves from P8-11 transgenic GFAP-EGFP mice were isolated intact and immediately placed in oxygenated *a*CSF at 37 °C for 30 min. Controls were incubated for a further 1 h in normal *a*CSF containing 10 mM glucose with 95% O_2_/5% CO_2_. Oxygen-glucose deprivation (OGD) was achieved using the method of Fern and colleagues [22], by incubating nerves for 1 h at 37 °C in glucose-free *a*CSF (osmolarity was maintained by replacing glucose with sucrose), and switching the chamber atmosphere to 95%N_2_/5%CO_2_. mGluR agonists ACPD and DHPG (100 μM) were added directly to the *a*CSF. At the end of 60 min normoxia or OGD, nerves were fixed immediately in 4% PFA for 1 h. Intact nerves were whole-mounted in vectashield and analysis by confocal microscopy. Images captured using a Zeiss LSM 710 Metaconfocal microscope, using a x40 oil immersion lens with high numerical aperture (1.3 nm), maintaining acquisition parameters constant between samples. Cells were counted within a constant volume of 20 × 20 μm in the *x*–*y*-plane and 15 μm in the *z*-plane, as previously described [23]. Data were expressed as mean ± SEM, where ‘*n*’ represents the number of nerves, and significance was determined by ANOVA and Newman-Keuls multiple comparison post-hoc analysis, using Prism 5.0 (Graphpad).

## 3 Results

### 3.1 Optic nerve astrocytes express mGluR5

**Fig. 1:**
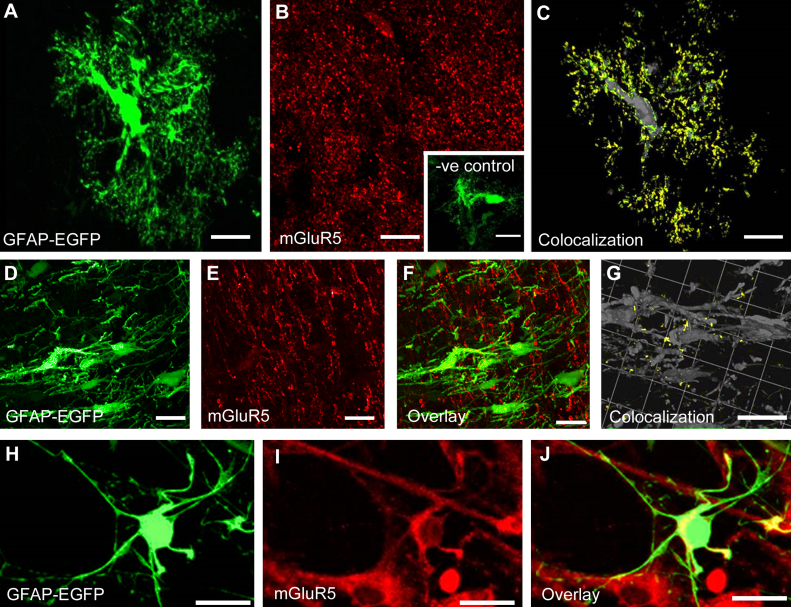
mGluR5 expression in astrocytes. Confocal images of brain sections (A–C), optic nerve sections (D–G) and optic nerve explant cultures (H–J) from GFAP-EGFP mice, to identify astrocytes (green) and immunolabelled for mGluR5 (red). (A–C) Cortical section illustrating protoplasmic astrocyte (A, green) and immunolabelling for mGluR5 (B, red), together with a colocalisation channel, in which voxels with the same intensity in red and green channels appear yellow (C, astrocyte appears grey); inset in (B) illustrates the absence of mGluR5 immunolabelling in negative controls in which sections were preabsorbed with antigen peptide. (D–G) Optic nerve section illustrating fibrous astrocytes (D, green) and immunolabelling for mGluR5 (E, red), together with the overlay of red and green channels (F) and the colocalisation channel (G, colocalisation of EGFP and mGluR appears yellow, astrocyte appears grey). (H–J) Optic nerve explant culture illustrating GFAP-EGFP+ astrocyte (H, green) and mGluR5 immunocytochemistry (I, red), together with the overlay (J, coexpression appears yellow), with evident mGluR5 immunostaining on the astrocyte cell somata and processes. Scale Bars = 20 μm.

Astroglial expression of group I mGluR5 is well documented in CNS grey matter, where they are important in synaptic transmission. Although glutamatergic signaling is also prominent in white matter, astroglial expression of mGluR was unresolved, and so we examined this in transgenic GFAP-EGFP reporter mice. Expression of mGluR5 by grey matter astrocytes was confirmed in cortical sections (Fig. 1A–C), and no immunolabelling was observed in controls in which sections were preabsorbed with antigen peptide (Fig. 1B, inset). Prominent colocalization of mGluR5 immunolabelling (red, Fig. 1B) and EGFP (green, Fig. 1A) was demonstrated by the generation of a colocalization channel (Fig. 1C, colocalization appears yellow and GFAP-EGFP+ astrocyte appears grey), using the negative controls to set the threshold and determine the voxels in which the green and red channels are of equal fluorescent intensity. Similar punctate mGluR5 immunolabelling was observed in optic nerve sections (Fig. 1E), some of which colocalized to GFAP-EGFP+ astrocytes (Fig. 1D and G). However, the optic nerve is densely packed with glia and myelinated axons and it was difficult to visualize cellular expression of mGluR5, which was achieved using optic nerve explant cultures prepared from P8 GFAP-EGFP reporter mice (Fig. 1H–J; coexpression appears yellow in Fig. 1J). The results demonstrate optic nerve astrocytes express mGluR5 on their cell somata and processes, but to a lower level than is apparent in grey matter astrocytes.

### 3.2 Group I mGluR evoke calcium signals in optic nerve astrocytes

**Fig. 2:**
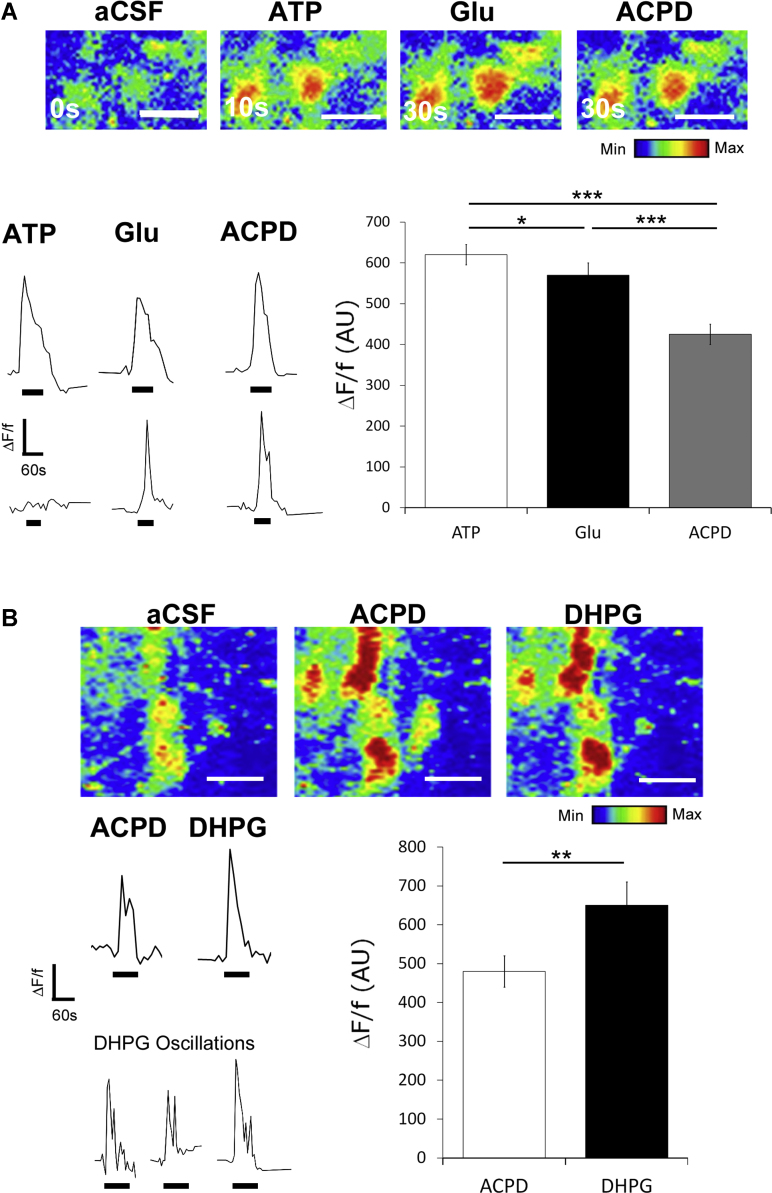
Group I mGluR evoke raised calcium in optic nerve glia. Optic nerves were isolated intact and loaded with Fluo-4 to analyse the response of glial cells to the group I/II agonist ACPD (A) and specific group I mGluR agonist DHPG (B). (A) Confocal images of Fluo-4 fluorescence intensity illustrated in rainbow false colour (upper panel) and representative traces of individual glia (lower left-hand panel) illustrate the response of glial cells to ATP, glutamate and ACPD (all at 100 μM and administered for 30 s). The bar graph shows the mean response (± SEM) expressed in arbitrary units (*n* = 41 cells from 5 nerves). (B) Confocal images of Fluo-4 fluorescence intensity illustrated in rainbow false colour (upper panel) and representative traces of individual glial cells (lower left-hand panel) illustrate that the selective group I mGluR agonist DHPG evokes a greater response than ACPD (both at 100 μM, applied for 30 s) and triggers Ca^2+^ oscillations. The bar graph shows the mean response (± SEM) expressed in arbitrary units (*n* = 13 cells from 4 nerves). Scale bars = 10 μm. *p < 0.05, **p < 0.01, ***p < 0.01, paired t-tests.

Stimulation of group I mGluR results in activation of PLC and InsP_3_-dependent release of Ca^2+^ from intracellular stores in hippocampal astrocytes [16], [17]. We examined this *in situ* in the isolated intact optic nerve from P8-15 mice, using Fluo-4 calcium imaging. The optic nerve contains mainly astrocytes and oligodendrocytes (>90% of cells), with minor populations of OPCs (<5%) and microglia (<5%) [24]. The bulk of cells that load with Fluo-4 in the intact optic nerve are astrocytes [19], [25], which were distinguished from other glia by the size of their somata; astrocytes have large somata (>12 μm diameter) that are separate from each other, whereas oligodendrocytes have small somata (<10 μm diameter) that are aligned in rows of 4 or more cells, and OPCs and microglia have the smallest somata that are isolated from each other by 20–50 μm [24], [26]. Recordings from cells with the features of astrocytes displayed robust and reproducible [Ca^2+^]_i_ elevations in response to ATP (100 μM), as reported previously, which was used to confirm glial cell viability at the beginning and end of the experiments [19]. Paired recordings in optic nerve glia demonstrate that individual cells respond to ATP, glutamate and ACPD, an agonist at both group I and II mGluR (Fig. 2A). The false colour images illustrate a typical response, with a mean relative potency of ATP>glutamate>ACPD (Fig. 2A; *n* = 41 cells from 5 nerves bar graph, *p* < 0.05, paired *t*-tests); in some cells the response to glutamate and ACPD was greater than for ATP (Fig. 2A, lower traces).

**Fig. 3:**
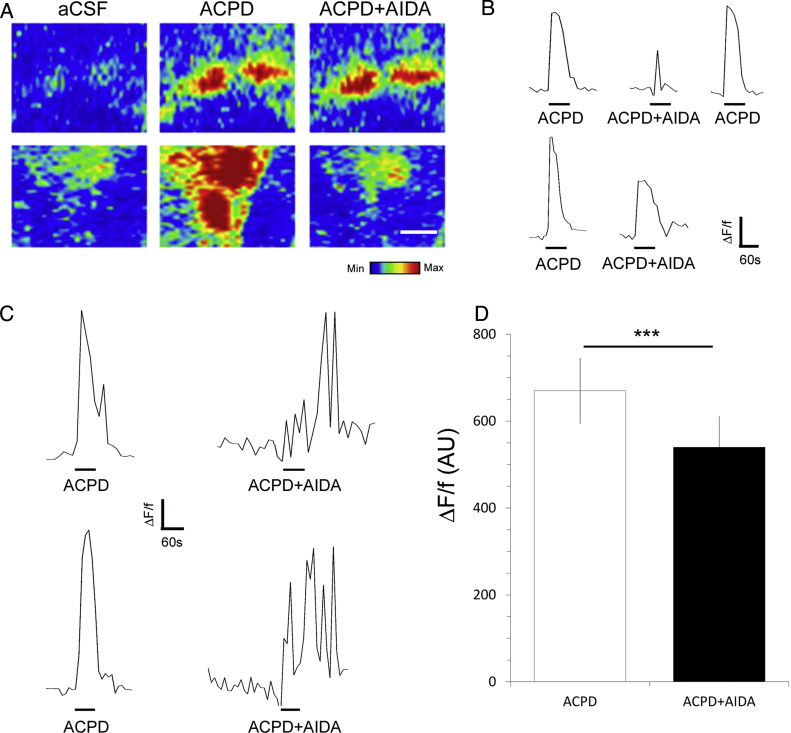
Group I mGluR antagonist AIDA significantly decreases glial calcium signals. Optic nerves were isolated intact and loaded with Fluo-4 to analyse the effect of the specific group I antagonist AIDA on the response of glial cells to the group I/II agonist ACPD. (A, B) Confocal images of changes in Fluo-4 fluorescence intensity (A, rainbow false colour) and paired traces from individual cells (B), showing that in some cells AIDA almost completely blocked the response to APCD, but in others only partially blocked the response to ACPD. (C) In many cells, Ca^2+^ oscillations persisted following blockade of group I mGluR. (D) Bar graph showing the mean response (± SEM) to ACPD was significantly less in the presence of AIDA, expressed in arbitrary units (*n* = 16 cells from 4 nerves). ***p < 0.001, paired *t*-test. Scale bar in *A* = 10 μm.

Paired recordings from Fluo-4 AM loaded optic nerves show that glia responded to the specific group I agonist DHPG (100 μM) with a mean maximum rise in [Ca^2+^]_i_ that was significantly greater than ACPD in the same cells (Fig. 2B; *n* = 13 cells from 4 nerves; *p* < 0.01, paired *t*-test). Notably, DHPG often evoked multiple Ca^2+^ spikes, characteristic of astroglial Ca^2+^ ‘oscillations’ (Fig. 2B, lower traces) [17]. The glial response to ACPD was significantly decreased in the presence of the specific group I mGluR antagonist AIDA (100 μM) (Fig. 3; *n* = 16 cells from 4 nerves; *p* < 0.001, paired *t*-test). The response to AIDA was variable and in some cells it almost completely blocked the ACPD response (Fig. 3A, B); this did not appear to be related to cell type and overall AIDA significantly inhibited glutamate-evoked Ca^2+^ signals in optic nerve glia. In addition, some cells displayed persistent Ca^2+^ oscillations following group I mGluR blockade (Fig. 3C), which has also been reported in hippocampal astrocytes in situ [17].

### 3.3 Group I mGluR protect astrocytes from ischemia in situ in the postnatal mouse optic nerve

**Fig. 4:**
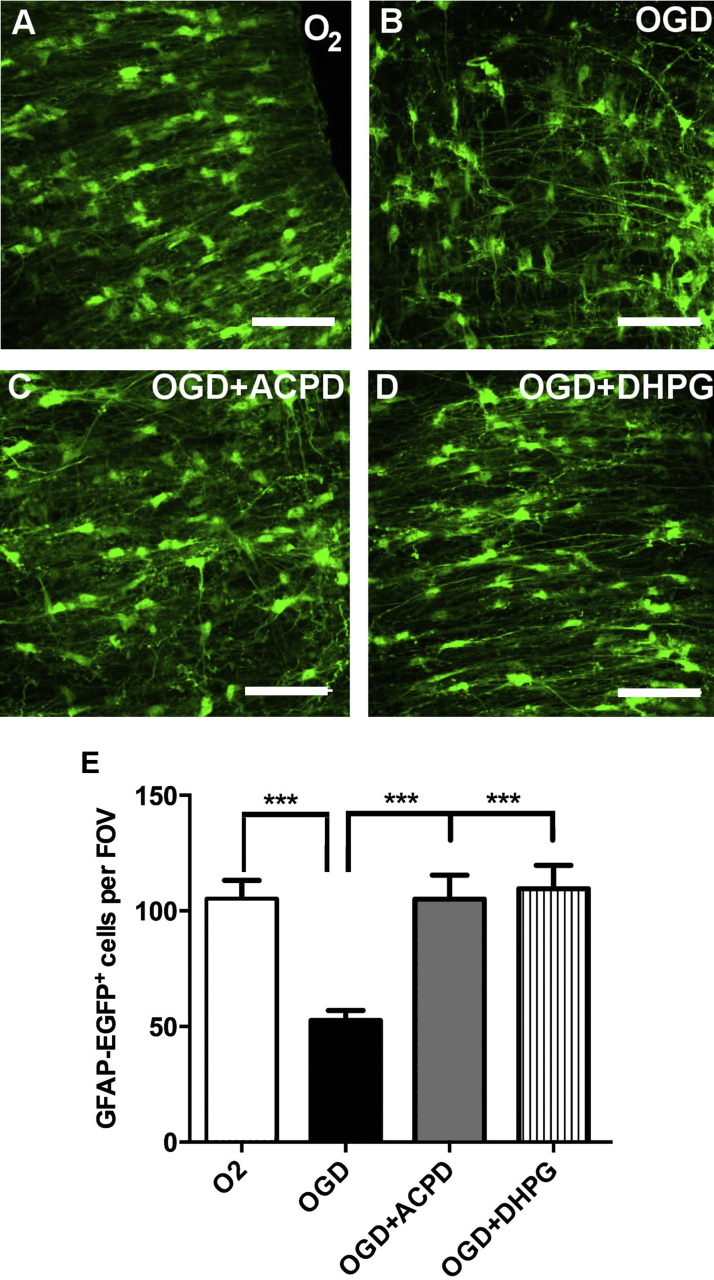
Activation of group I mGluR protects postnatal optic nerve astrocytes from ischemia. Optic nerves from P9 GFAP-EGFP reporter mice were maintained for 1 h in normoxic conditions with glucose (A), or exposed to 1 h oxygen-glucose deprivation (OGD), in *a*CSF (B), in the presence of the group I/II agonist ACPD (C), or the specific group I agonist DHPG (D). (A–D) Representative images of GFAP-EGFP^+^ astrocytes in isolated intact optic nerves; scale bars = 50 μm. (E) Bar graph of the mean (± SEM) number of GFAP-EGFP^+^ cells in constant fields of view (FOV; *n* = 5 nerves per experimental group; ***p < 0.001, ANOVA with Newman–Keuls multiple comparison post-hoc analysis).

Group I mGluR protect neurons from ischemia and their functional expression in the optic nerve suggests they may play a similar role in postnatal white matter astrocytes, which are highly vulnerable to ischemic damage. We examined this in situ in the isolated intact optic nerve from GFAP-EGFP mice aged P8-12, using the oxygen-glucose deprivation (OGD) model of ischemia (Fig. 4). Optic nerves were exposed to normoxic or acute OGD conditions for 60 min and analysed immediately, without reperfusion. The results demonstrate that optic nerve astrocytes are highly susceptible to ischemia, with a 50% loss after 60 min OGD compared to normoxic controls (Fig. 4A, B, E; *p* < 0.001, ANOVA and Newman-Keuls multiple comparison post-hoc analysis). Incubation with ACPD or the specific group I agonist DHPG almost completely protected astrocytes against ischemia, their number being significantly greater in ACPD and DHPG compared to OGD (Fig. 2A and C; *p* < 0.001, ANOVA and Newman-Keuls multiple comparison *post-hoc* analysis), and not significantly different than normoxic controls (*p* > 0.05, ANOVA).

## 4 Discussion

White matter astrocytes are highly susceptible to ischemia-hypoxia, with potentially devastating consequences for CNS function [2], [3], [4], [5]. There is evidence that group I mGluR are protective against brain ischemia and excitotoxicty in postnatal white matter in vivo [27], in situ in brain slices [28], [29], and in vitro in cultured neurons and astrocytes [30], [31]. Specifically, activation of mGluR5 has been shown to protect against neuronal loss in forebrain ischemia [32] and white matter damage in a rodent model of periventricular leukomalacia (PVL), the main cause of cerebral palsy and death in premature babies [27]. Here, we demonstrate that white matter astrocytes express mGluR5 and activation of mGluR5 protects astrocytes from ischemic injury postnally in situ in the mouse optic nerve. The results of our study indicate that targeting mGluR5 in astrocytes could contribute to an overall strategy for protecting CNS integrity and function in ischemia and other neuropathologies involving excitoxicity.

Our immunohistochemical evidence of mGluR5 expression in astrocytes is in agreement with studies that identified mRNA for mGluR5, but not mGluR1, in hippocampal astrocytes isolated from young and adult rats [33], [34]. Astroglial immunolabelling for mGluR1 was not successful in our hands, but has been detected by immunocytochemistry in 10% of cultured astrocytes prepared from spinal cord [35] and in a subpopulation of reactive astrocytes in multiple sclerosis lesions [36]. Our results indicated the overall level and pattern of mGluR immunostaining appeared similar in the cortex and optic nerve, although mGluR5 expression appeared greater in grey matter astrocytes compared to white matter astrocytes. Calcium imaging confirmed the functionality of group I mGluR in optic nerve glia identified as astrocytes on the basis of their large isolated somata [19], [26]; since mGluR1 were not detected it is likely this is due mainly to activation of mGluR5. In grey matter astrocytes, activation of mGluR triggers their release of glutamate and other gliotransmitters, which can evoke synaptic responses in neighbouring neurons and glia [37], [38]. Notably, glutamate is an important signaling molecule in CNS white matter [9]. Astrocyte processes contact axons at nodes of Ranvier [39] and respond to glutamate released during axonal electrical activity [19]. This would activate astroglial mGluR and potentially trigger their release of neurotransmitters, including glutamate, adenosine and ATP, which propagate intercellular Ca^2+^ signals between astrocytes and other glia [19], [40], to potentially modulate axonal activity and myelination [9]. Moreover, activation of mGluR stimulates the astroglial homeostatic functions of potassium and glutamate uptake [41], which would serve to couple astroglial homeostatic functions to axonal activity and help maintain axonal conduction, which is essential along long white matter tracts, such as the spinal cord, and for integrated cognitive function throughout the brain.

Activation of group I mGluR elicited Ca^2+^ oscillations in optic nerve glia, which is likely to be mediated by mGluR5, since mGluR1 tend to evoke single-peaked responses [42], [43], [44]. However, astrocytes can display various pattern of response, transient or oscillatory, depending on the species, brain regions and age of the animal [34], [45], [46]. In cultured astrocytes, activation of mGluR5 with ACPD mediates calcium oscillations via PKC phosphorylation [47]. In our experiments, DHPG evokes oscillatory responses, consistent with in situ studies on hippocampal astrocytes [16], [17]. ACPD does not evoke currents in white matter oligodendrocytes in brain slices [48] although it elicits a rise in [Ca^2+^]_I_ in cultured oligodendrocyte progenitor cells (OPCs) [49].

Astrocytes in the postnatal optic nerve were highly susceptible to ischemia, with a striking halving of their numbers after 60 min OGD, consistent with ischemia-induced apoptosis in astrocytes [3] and OGD-induced loss of immature astrocytes in the postnatal optic nerve [25], [50]. A key finding of our study is that activation of group I mGluR completely protected astrocytes in situ, which supports evidence that ACPD attenuates white matter loss in ischemia [14]. The influx of Ca^2+^ is one of the most significant events in ischemia and Ca^2+^ overload results in mitochondrial dysfunction leading to death [51]. In neonatal rat optic nerve, an important cause of astrocyte cell death is the run-down of Na^+^–K^+^ pumps and Na^+^–K^+^–Cl^−^ cotransport (NKCC1) during hypoxia/ischemia, which results in a rise in [Na^+^]_i_ and reversal of the Na^+^–Ca^2+^ exchanger (NCX) [52], [53]. In addition, membrane depolarization and subsequent opening of voltage-operated calcium channels (VOCC) results in a rise in astrocyte [Ca^2+^]_i_ initially through T-type VOCC within the first 10 minutes of ischemia, followed by L-type Ca^2+^ channels [25]. Interestingly, we show that activation of group I mGluR in astrocytes evokes a rise in [Ca^2+^]_i_, but this clearly does not induce cell death and is cytoprotective for astrocytes in ischemia. It is possible that mGluR activation alters NCX, NKCC or VOCC activity in astrocytes. Furthermore, increased extracellular glutamate is a major cause of excitotoxic cell death and activation of astroglial group I mGluR may indirectly reduce cell death by preventing a loss of astroglial glutamate transporters and thereby maintaining glutamate removal from the extracellular space [54]. Moreover, activated astrocytes upregulate the expression of group I/II mGluR [55] and specifically mGluR5 [56], [57]. These indirect effects are likely to be important in ischemia, particularly over the longer term, but an important cytoprotective effect of mGluR5 in astrocytes is likely to be direct protection against OGD-induced apoptosis, mediated through PLC and its effects on the PI3K/Akt, Nrf2 and NF-κB pathways [58], [59], [60], [61].

In conclusion, this study demonstrates that group I mGluR are cytoprotective for postnatal astrocytes in ischemia. Glutamate is an important signaling molecule in CNS white matter physiology and pathology [9], [10]. Our results provide new evidence that astroglial mGluR are important in these signaling cascades and represents a potential therapeutic strategy for limiting damage to postnatal white matter in pathologies that involve ischemia and excitoxicity.
